# The call of the unlived life: On the psychology of existential guilt

**DOI:** 10.3389/fpsyg.2022.991325

**Published:** 2022-09-29

**Authors:** Per-Einar Binder

**Affiliations:** Department of Clinical Psychology, Faculty of Psychology, University of Bergen, Bergen, Norway

**Keywords:** existential psychology, existential guilt, personal identity (the self), human potential, responsibility, vulnerability

## Abstract

This paper examines the psychology of existential guilt with Martin Heidegger and Rollo May’s conceptualizations as the point of departure. The concept of existential guilt describes preconditions for responsibility and accountability in life choices and the relationship to the potential given in the life of a human. It might also be used as a starting point to examine an individual’s relationship to the potential offered in their life and life context and, in this way, the hitherto unlived life of an individual. The following questions are discussed in contexts of identity development, perfectionism, and current cultural shifts in conceptualizations of selfhood: How can humans relate to the fact that only limited parts of who they might be can ever be actualized? Moreover, how can they relate to the fundamental ambiguity and “groundlessness” in the contexts of life where choices are made? There are striking parallels between the role of exploration in the Eriksonian approaches to healthy identity development and the ontological groundlessness that stands out as a premise for existential guilt. There are also parallels between identity fore-closure and normative identity styles and “falling” into das Man in the existential framework. Perfectionistic ideals easily become an objectivization – and closure – of possible alternatives and choices. In contemporary theories of constructions of selfhood, the dangers of alienation from the community on the one hand and escape into what might become totalitarian collectivism on the other is pointed out. The contextualization of personal responsibility offered through the concept of existential guilt might address both dangers. It provides a perspective on how personal responsibility is embedded in contexts of human relationships, relationships to nature, and the finitude, freedom, uncertainties, and suffering that is given through human existence. Existential guilt can wake sorrow and regret over opportunities overlooked and lost. However, most of all, it can be seen as a drive toward repair in the relationship toward both oneself and the other. It takes the form of receptivity, an openness to a life not yet lived, and creative use of imagination.

## Introduction

The lifetimes of humans are very limited, considering possible choices of how to live a life. Nobody fulfills all their potential. How can humans relate to the fact that only limited parts of who they might be can ever be actualized? Moreover, how can they relate to the fundamental ambiguity and “groundlessness” in the contexts of life where choices are made? Here, I will use Heidegger’s (1957) conceptualization of existential guilt to examine the psychological implications of being responsible and accountable for one’s life on an ontological and fundamental level. Within the frame of Heidegger’s ontology, guilt is given as an ever-present and unavoidable part of human existence. With Heidegger’s conceptualization as a starting point, I will examine the concept of existential guilt as it has been used by May (1958) within humanistic-existential psychology. In this context, the concept of existential guilt is strongly related to the individual’s responsibility for actualizing their potential. Differences between Heidegger and May’s conceptualizations will be pointed out, alongside the discussion of the need for a conceptualization of existential guilt in our understanding of the psychology of authenticity and responsibility. The dilemmas related to death and finitude, the role of regret on the one hand, and the pressures toward conformity on the other will be discussed. I will also explore existential guilt and responsibility concerning the explorative aspect of identity development (Erikson and Erikson, 1998; Berzonsky, 2004; Kroger and Marcia, 2011). A question related to this is how we can differentiate between existential guilt and current perfectionistic ideals of becoming “the best version of myself” (Negru-Subtirica et al., 2021). Cultural shifts in how selfhood is to be understood may bring new possible meanings to existential guilt as a phenomenon. Therefore, I will discuss the possible role of existential guilt in critical analyses of the neoliberal self-reliant self and of what is recently described as “victimhood culture” (Bauman, 2013; Campbell and Manning, 2018).

From Heidegger’s (1996) point of view, we are always and necessarily guilty—we exist in a way that implies that we are constantly accountable to ourselves. Within the framework of humanistic and existential psychology, the concept of “existential guilt” is mostly used in another way; to describe a specific feeling that arises when human beings fail to live as fully as they can (Yalom, 1980). However, both within Heidegger’s thinking and humanistic-existential psychology, the concept of “existential guilt” is to be understood on the basis of the assumption that there is no pre-given legitimation or justification for our decisions. Our most profound choices are made when we allow ourselves to experience what Kierkegaard (2013) describes as “the dizziness of freedom” (p. 61), a state of mind that he also characterizes as “existential anxiety.” Yalom (1980), building both on Kierkegaard, Heidegger, and May, describes this lack of pre-justification for choices as “groundlessness.” Yalom points out that one can differentiate existential guilt from the guilt that arises from transgressions toward others and neurotic guilt that arises from imaginary transgressions. Failing to relate to and actualize one’s potential—existential guilt—is seen as a third form of guilt with broad existential implications. Existential guilt, in this sense of the word, both differs from how Heidegger uses it and bears some similarities. May and Yalom describe it as a specific psychological phenomenon. And they do so in a way that, in contrast to Heidegger, is closer to a romantic notion of an individual self-expressing self in search of wholeness and completeness. Heidegger’s ontological analysis may be an important point of departure for problematization of this notion, and also may have important implications of how we can understand the psychology of human life-choices.

Inspired by Kierkegaard (1946, 2013), Heidegger (1957) describes guilt, in its fundamental and existential sense, as a prerequisite for living a life that rightfully could be called “one’s own,” and by this to live in “Eigentlichkeit.” Existential guilt, in Heidegger’s sense of the word, as an ever-present existential given, calls upon us to be answerable and accountable to ourselves, for ourselves. According to Heidegger, this fundamental sense of accountability is a precondition for ordinary moral guilt. Within psychology, Heidegger has often been read through existentialist lenses. In existentialist interpretations of existential guilt, such as Sartre (1992), the central distinction has been set between authenticity and the “bad faith” of choosing not to choose by giving in to the mere conventional outlooks of life. However, in Heidegger’s (1996) own conceptualization of existential guilt, he describes a more complex relationship to the “they,” or, in German, “das Man,” which is our ordinary, socially constructed, and conventional ways of meaning-making. We cannot make a complete break with “das Man.” These shared frameworks of meaning will always be an essential part of how we understand and orient ourselves in the world. Authenticity becomes possible through “existential modification” (p. 130) of das Man. Through such modifications, we become ready to give voice to our experience and take action based on the particular aspects of our life situation (Wrathall, 2015). Our shared understandings of the world allow us to see ourselves from a more general perspective as “anyone” in our social group. Some fusion between our own thinking and the shared frameworks of the group is necessary for our functioning as a social species. However, it comes with side effects; we can become absorbed into the general and non-specific perspective on life in the way we understand ourselves. We might become alienated from the unique practical and relational circumstances—and obligations—in our lives. Existential guilt, then, call us back to a life that is truly our own and to respond to – rather than passively follow – das Man. Authenticity becomes possible when a person “explicitly discovers the world and brings it near” (Heidegger 1996, p. 129), and thereby finds a way to position him or herself toward the specific circumstances this person is “thrown” into. In this way, authenticity can be seen as a prerequisite for true responsibility.

Within the framework of humanistic-existential psychology, May (1958) describes this form of guilt as an inner warning or reminder that one is not utilizing one’s potential. He also relates existential guilt to situations where one fails to fully perceive one’s responsibilities concerning other people and the natural world. May’s use of the term guilt is ambiguous – he also discusses it as an intrinsic part of human existence and an ever-present call for responsible action. However, May (1983) and Yalom (1980) often also use the concept of existential guilt to analyze clinical situations in psychotherapy, specifically where persons deny specific potentialities and responsibilities in their lives in ways that cause psychological suffering. In Heidegger’s (1996) terms, this can be described as analysis on an ontic level – a study of concrete beings and the contingent conditions in their life situations – rather than an ontological level that relates to the deeper structure of Being, and our accountability to ourselves at a fundamental level. This paper will explore some ontic psychological implications of Heidegger’s ontological view relating to our experience of authenticity and responsibility, and in connection with this, time and temporality.

## Heidegger’s perspective on existential guilt and our lifetime as persons

“Being-in-the-world” is always an engaged type of being, according to Heidegger (1996). He analyzes how we position ourselves in our world through a deep existential structure of “Care.” Care has three dimensions: “facticity (thrownness), existence (projection [of possibilities]), and falling [into the ‘they’]” (p. 284).

The dimension of “thrownness” describes the contextuality and situatedness of our lives (Heidegger, 1957). We must constantly relate to what is already given. We are thrown into circumstances that we have not chosen, but also with decisions that we have already made, and at least some of them we regret. Factual reality is always already given in our life. However, the world we live in is, at the same time, fundamentally ambiguous and open to a wide range of interpretations. Therefore, we are at the same time bound by what is given through our “thrownness,” and free to relate to, shape, and give meaning to our destiny.

The dimension of “existence” describes the role of projection of possibilities (Heidegger, 1957). We are forward-leaning beings. The choices we make always point toward possible futures. How we project meaning into our future will also shape our awareness and interpretations of our presence and past. In his existentially oriented novel “Life is elsewhere,” Kundera (2000), an author strongly inspired by Heidegger, describes a situation where the protagonist’s mother chooses a partner. It is a choice of a future path that evokes ambivalence in her. Her decision makes her see her past in another hue than before:

“Do you think that the past, because it has already occurred, is finished and unchangeable? Oh, no, it is clothed in mutable taffeta, and whenever we look back at it, we see it in another color” (p. 88).

It is as if another past emerges, where new aspects of memories are brought into the foreground, and she also relates to somewhat other memories than before. She is living her adult life, neither young nor old, and is ambivalent about whether she wants change or continuity in her life. Her choice reflects that she prioritizes continuity and stability out of concern for her son, Jaromil.

The third dimension that Heidegger (1957) analyzes, “falling,” points toward the always alluring possibility of being absorbed into the social conformity of das Man. Das Man is more than a blind following of social conventions and norms. It is also being taken away by thoughts, assumptions, and ways of understanding that we borrow rather than own. When Heidegger analyses how we are carried away by das Man, he uses the word “falling.” He thereby uses the metaphor of a physical process to point out how we are easily absorbed into the “they.” As Carman (2007) points out, Heidegger uses the distinction between authenticity and inauthenticity in both a descriptive and evaluative way. At a descriptive level, “Falling” into das Man and some degree of inauthenticity is given as part of human existence. It is a prerequisite for everyday functioning and understanding of ourselves and others. In normative contexts, Heidegger seems to draw on a threefold distinction between authenticity, inauthenticity, and an “undifferentiated” average everydayness. A flexible flow between our individual lived experience, and the shared social perspective is part of our ordinary functioning as individuals (Carman, 2007). However, constantly interpreting one’s life and possible actions through the lenses of how anyone makes meaning to it puts us into a state of alienation and disowning of the specific possibilities that could have been truly our own. This inauthentic interpretation of oneself as an anonymous part of das Man, can be a way of disburdening oneself with the responsibility for one’s own existence. This type of inauthenticity might feel like a natural state until existential anxiety arises and shatter our taken-for-given assumptions about ourselves and our world. Kundera (2000) gives an example of the existential dilemma recognizing this “fallen” aspect of thought and language might rise as the protagonist, a young poet, discover his lack of originality:

“Jaromil was no longer at all convinced that everything he thought and felt was solely his, as if all ideas had always existed in a definitive form and could only be borrowed as from a public library. But who then was he? What could his own self really consist of?” (p. 29).

For Jaromil, the insight into how his thoughts about life and existence were not originally his own, in some sense, wakes him up. But it also stirs up uncertainty with many of the typical traits of an identity crisis in young persons and a search for values, roles, and new ways to construct his self (Erikson, 1968).

The connotations of Heidegger and Kierkegaard’s concepts are somewhat different from the English concept of “guilt” and are closer to being responsible in the broader sense. Heidegger (1996) proposes that “Dasein is essentially guilty” (p. 353). The German word for guilt is “Schuld.” Like the Scandinavian verb “skylde,” “schulden” also means to owe something to someone. We owe a responsibility to ourselves and others. This is true even when we are bound to relate to the circumstances we are “thrown” into. We are arbitrarily born into a particular historical epoch, a family context, and certain bodily and psychological dispositions as our starting point. Indeed, some of these circumstances may be painful, but we must relate to them to get ownership to our lives. Heidegger (1996) points out how we must start to “own” our starting points: In Being-guilty, Dasein “must take over Being-a-basis” (p. 330) into its own existence by “project [ing] itself upon [the] possibilities into which it has been thrown” (p. 330). This “Being guilty” is an essential characteristic of what Heidegger describes as “resoluteness,”; the authentic existential attitude of the self to itself, with an anticipatory and future-directed commitment to one’s life. In this commitment lies a realization that our lives as a whole, with our present activity included in it, and a well of other engagements and responsibilities, are not infinite. “Being guilty” is only to be understood “in so far as Dasein discloses to itself its potentiality-for-Being, and discloses it ‘right to its end.’” (Heidegger, p. 353).

According to Heidegger’s (1996) conceptualization of guilt, a true commitment in our lives depends upon anticipation of finitude and authentic Being-toward-death: “As Being-toward-the-end which understands—that is to say, as anticipation of death—resoluteness becomes authentically what it can be.” (p. 353). According to Heidegger, finitude individualizes us, as one can only die one’s own death. When we realize that our death is real, personal, and absolute, we also become ready to make our own modifications to the “they.” The young and idealistic protagonist in Kundera’s (2000) novel formulates how his alter ego, Xavier, a phantasy figure, wants this insight to be the foundation for all his everyday actions:

“He detested the pettiness that made life semilife and men semimen. He wished to put his life on one of a pair of scales and death on the other. He wished each of his acts, indeed each day, each hour, each second of his life to be measured against the supreme criterion, which is death.” (p. 70).

The idea of living every minute with death-awareness is, of course, neither realistic nor desirable; some actions should be allowed to be mundane. Existential awareness will necessarily be something that ebbs and flows. And a hint of narcissistic superiority is easy to recognize in the daydream of the protagonist. In some way, it also stands out as a parody of the Heideggerian view. However, the constructive function of death awareness also rings true; how it can call us back to our own lives and let us acknowledge our lifetime and the limited contexts of our lives as our actual possibilities. For Heidegger, “death” is not most and foremost a specific event but an aspect of finitude and transitionality that are part of all human experience.

Heidegger (1996) describes that conscience “calls” us back from das Man, and when “Understanding the call, *Da-sein listens to its ownmost possibility of existence*. It has chosen itself.” (p. 287). Later, he specifies, “What is chosen is *having* a conscience as being free for one’s own most being-guilty” (p. 187). Understanding the call means becoming accountable to oneself for oneself. Stepping out of the lostness of das Man also means facing existential anxiety: We realize the fundamental lack of outer foundation for our choices, as we live in a world that is at the same time ordered and understandable, fundamentally ambiguous, and thereby open to new meaning. We are not identical with the sense of self that das Man offers us. Das Man mainly makes us see ourselves as a representative of “anyone.” Enigmatically, Heidegger (1996) describes existential guilt as “being-the-ground for a being which is determined by a not, that is, *being-the-ground of a nullity*” (p. 283). One way to interpret this is that we recognize that we can never wholly lean on norms and interpretations of life given by das Man. We become less of ourselves if we let ourselves be entirely determined by the anonymous construction of our everyday world offered by das Man, and mainly take up the project the situation poses on us; this way of living may give a fragile illusion of solid ground, but do not allow us to fully inhabit our world (Wrathall, 2015). When we recognize “nullity” or “groundlessness, we can truly engage in the particular style of inhabiting possibilities that characterizes our life situation, and with this as a starting point, take responsibility for it. We do not do this by stepping out of das Man; then, we would not have any system of meaning at our disposal. We make our own “existential modification” of das Man, and in this way, we act within the frame of the particular circumstances of the life that we can call “our own” (Heidegger, 1996). When we recognize our groundlessness in das Man, our lostness starts to matter to us. It is no longer an option to legitimize one’s choices upon the impersonalized normative statements of what “one” does. This dialectic between dependence on systems of meaning and the need to create our own meaning bear some similarities to what Winnicott (1971) described when he said that “it is not possible to be original, except on the basis of tradition” (p. 99).

Facing this uncertainty and vulnerability, we make choices grounded in our own potentiality-for-Being.: “Wanting-to-have-a-conscience becomes a readiness for anxiety.” (p. 342). When we take a step back from das Man, we also step into a vulnerable state. Our choices always point back on ourselves as the one who has chosen. Choices are always made in an ambiguous and, therefore, always potentially anxiety-provoking context. As Kierkegaard (2013) points out, ambiguity might be a source of both anxiety and creativity. We have no other solid ground than what we create through our commitments. However, Heidegger’s “existential modification” of das Man does not disconnect us from social norms. How are we to understand an authentic way of relating to norms? One way to analyze this, is to interpret guilt in the existential sense as a call to evaluate, ask for – and give – reasons for how to relate to existing norms in the social world we inhabit (Crowell, 2013). A different interpretation is to understand existential guilt as a sensitivity to the particular in one’s situation and a readiness to take action based on that, with an openness and accountability that goes beyond norms but are not antithetical to them (Wrathall, 2015). This readiness is partly founded on what is given through the “Befindtlichkeit” or “disposedness” in our life situation, with its specific emotional significance, our specific character traits, wishes, and dispositions. It is also based on our ability to “project onto possibilities” and see the possible significance of what is given in our life situations in light of opportunities for actions. In this way, my “practical identity” is more than my social role and given dispositions (Wrathall, 2015); I also define myself in light of more opportunities for action than I can ever pursue. In this way, when I make choices, I also have to take over the “groundlessness” given through existential guilt.

## Rollo May and situated existential guilt in a psychological sense

May’s (1958) theory of existential guilt is clearly inspired by Heidegger’s philosophy but formulated mainly within the framework of humanistic-existential psychology. For May, existential guilt—or what he calls “ontological” guilt—is a quality in how one relates to one’s potential; it is part of the “Eigenwelt” of the individual. May’s use of the term “ontological” in this context is not correct, or it is at least very different from Heidegger’s use of the term: May describes a specific psychological and, therefore, “ontic” phenomenon, not an ontological condition. This form of guilt that May describes is a constructive emotional state; it calls us to examine taken-for-given assumptions about ourselves and our relationship to the world, exploring potential ways of being. In this way, and compatible with certain interpretations of Heidegger’s thinking (e.g., Wrathall, 2015), authenticity can be seen as a quality in the way that we integrate our disposedness and our projects within the world we inhabit. In May’s view, we can repress important potentials, and the repression of potentials is a source of inauthenticity. For May (1983), the unconscious mainly consists of such potentials; he defines it as “those potentialities for knowing and experiencing that the individual cannot or will not actualize” (p. 18). Which possibilities that are repressed may be due to the forces of das Man, and what current society finds worthwhile and acceptable when we become subject to external demands, rather than being intrinsically motivated by social ideals. The repressed will also have its origin in ways of judging oneself with roots in a family of origin and interaction between parent and child during early years when parents put outer demands on a child without emphatic immersion with the child’s emotional, motivational and characterological disposedness. May stays close to a Sartrean interpretation of authenticity, as an antidote to das Man. May was a critic of certain bureaucratic and technocratic aspects of modernity and their possible damage to the individual’s inability to understand their authentic selves. His views on the role of self-expression in authenticity in some ways differ from Heidegger’s view on the always unaccomplished construction of the self and are closer to the romantic notion of authenticity as a form of individualistic completeness and self-realization (May, 1994; Blattner, 2013; Abzug, 2021).

However, for May, the potentials of an individual, and the responsibilities and commitment that follow when potentials are fulfilled, must be understood in a wider context than the individual in isolation. Human potentials are fundamentally dependent upon how the individual’s life is embedded in the destiny of others, our “Mitwelt” (May, 1958). May points out how we will never fully grasp the “otherness” of others, and therefore unavoidably tend to “do” violence to the true picture “of others,” and to understand others and their needs through our own “limited and biased eyes” (p. 115). In a healthy state of existential guilt toward others, we, therefore, owe them an attitude of humility and forgiveness. Here, May is in line with Buber’s (2003) statement that “Only in partnership can my being be perceived as an existing whole” (p. 53). It is also very much in line with de Beauvoir’s (1962) idea that our personal freedom is only possible through the freedom of others. Only others can make us extend our imagination and reflection beyond our own life: “To make being ‘be’ is to communicate with others by means of being.” (De Beauvoir, 1962 p. 71). Our potential as persons is fundamentally interwoven in our ability to form relationships with others.

May (1958) also briefly outlines existential guilt in our relationship to nature, our “Umwelt.” He describes this as a form of “separation-guilt” in relation to nature as a whole and links this to our Western scientific worldview. He does not explicate this, but it seems reasonable to see this in connection with Heidegger’s (1977) view on how a purely “technological” stance toward nature (and ourselves) has the potential to alienate us, a line of thinking that has been further developed by Abram (2012) in recent years.

## A call for responsibility or a call for growth?

Heidegger (1957) and May (1958, 1983) highlight somewhat different but often compatible aspects of our responsibilities toward our existences. For Heidegger, the crucial axis of conflict is between the always alluring but dangerous possibility of becoming unreflectively absorbed into das Man versus the possibility of being accountable to oneself through modifying it in one’s own way. Authenticity can be seen as living truly in a truly explorative relationship to our disposedness and embracing the particular details of our life situation, and at the same time being open to taking action in a way that takes into account that our range of possibilities is always bigger than our actual selves (Wrathall, 2015). Our relationship to our finitude and the fact that one day we will die potentially wakes us up from the blind following of das Man and leads us to recognize the depths of our responsibility. Awareness of death individualizes us and makes us realize our life as our own. Although our possible choices are unbound and unlimited, our commitments get their meaning as we recognize that our time, and by this, the number of possibilities we can actualize, is limited. These are also important issues for May. However, as a clinical psychologist, he is working on an ontic rather than an ontological level. He saw the individual’s ownership of the potential given in their life versus denial of ownership as a central conflict and creative self-expression as a means to get to know these potentials, that in this way, may be examined in relation to self, others, and nature. May is very much in line with what Becker (1973) describes when he states that guilt is something that “results from unused life, from ‘the unlived in us’” (p. 180). Not only our life as it is but our unlived life as well shapes and forms our destiny.

There are parallels between romantic aspects in May’s view of human potential and Maslow’s (1962) idea of “self-actualization” in humans. However, this is not so much of an individualistic view as it may seem through contemporary lenses. Today, self-actualization has connotations of self-absorbed strivings due to how self-help culture has adopted the concept. However, as pointed out by Kaufman (2021), social responsibility and self-transcendence were essential premises in the original version of this concept. In Maslow’s theory, the distinction between “deficiency” and “growth” motivation is central. Suppose we are to connect the idea of existential guilt to this framework. In that case, we might say that in a humanistic approach, existential guilt is also a specific responsibility for taking care of one’s growth as a person and as a relational and ecological being. Heidegger (1996), on the other hand, points out the unavoidability of letting go of certain possibilities is an essential aspect of our existential guilt. On the ontological level, we are already “guilty” toward the possibilities within the circumstances we are thrown into. When we authentically discover and find our way to position ourselves within these circumstances, we also relate to the fact that we are thrown into particular possibilities rather than others. Responsible ways of choosing which possibilities to follow also mean letting go of other possibilities. This deeper sense of responsibility given through existential guilt can be seen as a precondition for actualizing potentials. If you, for instance, are “thrown” into the world of parenthood, letting go of the carefree world of being a bachelor, or very dedicated party-animal, is a precondition for actualizing your potential as a good parent. Existential guilt is first and foremost about relating to one’s actual life. The fulfillment of certain potentials will always be specific to a life context where other potentials must stay unused. Heidegger’s perspective of authenticity is therefore incompatible with any romantic idea of “complete” self-actualization (Carman, 2007).

## Existential guilt and temporality

Especially in Heidegger’s (1957) conceptualization of guilt, time and temporality play an important role. In this analysis, finitude personalizes us. We experience events and actions in our life with an awareness that our time and possibilities are limited. Neither Heidegger nor May explicitly discusses the feeling of regret in relation to existential guilt. Considering that opportunities for actualizing one’s potential in life unavoidably will be limited—can recognizing lost opportunities, and thereby regret, be seen as a vital part of existential guilt as a phenomenon?

Contrafactual thinking about one’s life allows one to explore life’s complexities and thus contribute to the experience of meaning (Kray et al., 2010). The realm of “what might have been” can awake feelings of gratitude. However, it also, not seldom, awakens regret. Reflecting on regrets and lost possible selves is an unpleasant activity but is concurrently related to complexity, richness, and meaning in self-experience (King and Hicks, 2007). Regret is the negative emotion people value most; it gives us insight into past actions and current dispositions and prepares us to engage in approach and avoidance behaviors (Saffrey et al., 2008). What Heidegger discusses as the “existence” dimension of existential guilt implies responsibility for what might become the future. Regret, combined with this forward-leaning sense of responsibility, can mean that we can fuse our imagination of “what I wished I had done (or not done),” “what might have been if I had done so” with “what might still become possible if I act differently from what I did.” The call for responsibility for one’s life, and the call from the hitherto unlived life, not seldom arise from regret.

How will awareness of death-and by this, the absolute limit of time – influence how we make our choices? Research in the Terror Management Theory tradition (TMT; Pyszczynski et al., 2015), with its theoretical base in Becker’s (1973) work, emphasize how we defend against death awareness: We develop cultural worldviews that provide meaning, order, and coherence to existence in a way that offer us standards for actions that might increase our self-esteem, and outline worth and grant symbolic immortality through participation in religious or social institutions. Suppose we are to follow the findings from this research in the TMT tradition. In that case, we might say that this automatized tendency to act defensively, that death-awareness pushes us into being conformist followers of das Man rather than making authentic and individualized choices. The tendency to repress our mortality is strong, and the result of such repression goes in the direction of what we would predict from Heidegger and May’s views of death denial. However, suppose their views on the role of acceptance of finitude in existential guilt also is true. In that case, death awareness without denial must also be possible and result in a heightened awareness of choices and freedom. In several studies, death awareness seems to draw us in the opposite direction than being a conformist follower of das Man, as shown in studies of posttraumatic growth and studies of reactions of persons narrowly avoiding death (Joseph and Linley, 2005; Wu et al., 2019): Confrontation with death also often lead people to reevaluate their values and to explore and create new meaning regarding the self, others, and the world.

From a life span perspective, death will have a different place on the horizon of the young and the old. How will the desirability of making new choices and actualizing potential change? An entry into this question is studies of autobiographical reasoning. Younger persons often focus on change in their autobiographical reasoning; older people emphasize exploring coherence (McLean, 2008). The young will be motivated by possible future possibilities; for them, change is possible and desirable. Older people will to a more substantial degree, be motivated by an attempt to maintain a coherent and continuous sense of self in the threats of loss and life changes. However, both groups seem equally likely to engage in life reflection (Staudinger, 2001). Constructing and creating new meaning to temporally organized experiences of who we are, have been, and will become will be ongoing as long as we live. Actualizing potential and acting authentic can also be an inside job; it can take the form of a revision of how one relates to oneself and understand life and its values. Heidegger (1996) also discusses positive emotional qualities in how we relate to our potentials as a reality alongside anxiety: “Together with the sober Angst that brings us before our individualized potentiality-of-being, goes the unshakable joy in this possibility (p. 310).” At the deepest level, realizing potential is more than a job; it is a way of being. Indebtedness is only one side of the coin in an authentic response to what the world offers. In his later writings, Heidegger (1968) describes “thanking” and grateful thought as something that might arise when we allow ourselves and the world to be as it is and think with a quality of openness and awe. This implies seeing the existence of ourselves as a gift and feeling grateful for being amid beings that present themselves to us, not to manipulate or exercise power over them, but just to let them be.

## Existential guilt and conformism

Although our relationship and the Mitwelt can be seen as the arena of our most profound existential responsibilities, the social world is also the arena of das Man, and the possibility to be unreflectively driven away about conventional beliefs about life and life choices. It is as if das Man has its own gravity; it often feels like following the path of least resistance. Why? How are we to understand the relationship between existential guilt and conformism?

As Fromm (1941) points out, blind following of conformist ideas and norms can be a “flight” from personal freedom that shelter us from realities of life that have to do with existential vulnerabilities and limitations. Sunstein (2019) analyzes how conformism can result from group pressures, polarization, and prioritizing belongingness over free thought and choices. And, as pointed out in the prior paragraph, the tendency to prioritize belongingness and belief in systems of meaning can be seen as a defense against death awareness (Greenberg et al., 2014).

Another psychological reason why we fall into das Man may be in line with the functional aspects of conformity that Sunstein (2019) also describes; there is often accumulated wisdom in conventional thoughts, assumptions, and choices. Choosing to marry, have a child or two, buy a house in a suburb, getting a Golden Retriever and a Japanese car may be a way to set the practical scene for a safe, structured, and harmonious life. It is a choice that can easily be made without much reflection and awareness because it is a norm in many social groups. However, making a choice that fulfills the norm in your social group does not have to be motivated by the pressure to conform or the tendency to blindly “fall” into das Man. A highly ordinary setting can also set the scene for a rich and meaningful life, chosen with a deep awareness of finitude and existential responsibility. In the stereotype of existential thinking, an authentic life is often depicted as an unconventional life. However, although conventionality may make a flight from existential guilt more easily go unnoticed, the “resoluteness” and “existential modification” that Heidegger (1957) describes seems more related to a quality of awareness and ownership in how one chooses than to external characteristics in what is chosen. Choosing to live according to conventional ideals can be done with authenticity, although obeying conventionalism as an outer demand cannot so.

Conventionalist subordination under Das Man as a way of being can be seen as a disengaged form of meaning-making, a distanced way of talking about realities of life. Life choices, values, and even death can be handled as if these realities were nothing personal as if they belonged to no one in particular, and least of all, oneself (Heidegger, 1957). Acting as a nonconformist, a seemingly creative rebel, can be a way of hiding such an inner state from oneself and others just as much as being an anonymous bureaucrat following the movements of their organization. There is an essential quality of lostness in this state, which Kierkegaard (1946) describes as “despair,” and where a personal sense of obligation and presence is lacking. Although conformity more easily makes our lostness go unnoticed, nonconformist choices are not in itself a solution.

## Existential guilt and formation of identity

Our choices and commitments will, in some sense, answer the “who am I?” question and be part of constructing personal identity. How are we to understand the relationship between existential guilt and personal identity?

From a developmental perspective, existential guilt may be seen as a call for discovering and creating an autonomous personal identity. Erikson (1968) uses the concept of “identity crisis” to describe periods in a person’s life where values and choices are reevaluated and reexamined. Erikson specifically examines this in connection with adolescence and young adulthood, but not exclusively so. Personal identity needs to be reconsidered, rediscovered, and recreated as roles, commitment, and bodily status change throughout life (Erikson and Erikson, 1998).

James Marcia and colleagues (Marcia, 1966; Kroger and Marcia, 2011) base their empirical studies of identity development on Erikson’s theory, where “exploration” and “commitment” are two central concepts. What they describe as “achieved identity” depend upon a commitment to values, ideals, roles, and relationships. This achievement is only possible after a period of exploration of potential choices, both in breadth and depth, which Erikson (1968) describes as a “moratorium.” In this view, healthy identities will always be open to further exploration. As Schachter (2005) points out, Erikson’s concept of exploration can be seen as a broad intrapsychic and transactional deliberation of identity.

In line with this emphasis on exploration, Berzonsky (2004) examines the epistemological dimension of exploring potentials and making commitments. He describes people with an “informational” identity style as actively seeking to understand their life context, and the values, roles, and relational commitments that may be building blocks for personal identity. They are ready to reevaluate knowledge relevant to ideas of who they are and operate with an epistemology where ambiguity in life and choices made are recognized and tolerated.

In these identity theories, the non-optimal but still functional types of identity formation – what Erikson described as “identity foreclosure” and Berzonsky describes as a “normative” style – are built upon conventionality and passively adapting to socially prescribed roles and values and are linked to authoritarianism (Berzonsky and Adams, 1999; Ryeng et al., 2013).

There are striking parallels between the role of exploration in the Eriksonian approaches to healthy identity development and the ontological groundlessness that stands out as a premise for existential guilt. There are also parallels between identity fore-closure and normative identity styles and “falling” into das Man in the existential framework. However, the aspect of finitude and the individualizing power of death-awareness is a topic in these theories only to a more limited degree.

According to Erikson’s (Erikson and Erikson, 1998) theory, “despair” because of unused potential can occur in a late stage of life. At this stage, it is more of a state of resignation than a possible awakening and motivating force. However, the middle of adulthood is associated with an increasing understanding that life is, hopefully slowly, heading toward an end. Awareness of this reality leads to either the development of generative ways of acting for the benefit of future generations, or to stagnation, with the cessation of active contributions as a citizen of society. Although not so often explicitly discussed, the constructive role of a certain level of uncertainty, anxiety, guilt feelings, and conflict, and therefore, the potentially painful dimension of exploring potentials and choosing commitments, lies implicit in theories of identity formation. Erikson (1968) used the term “identity crisis” to describe a conflictual element connected with questioning the sense of self and its foundations. The current need for such a non-medicalized concept of normal identity crises has recently been pointed out (Côté, 2018). A further discussion of the existential underpinnings of identity and an increased awareness of the normal struggle involved in identity formation is needed.

One of the implications of the concept of existential guilt on a psychological level is that taking ownership of one’s life and its potential also is an anxiety-provoking process. On the one hand, on a cultural level, there has been a trend toward medicalization of a too broad range of human suffering in recent years (Binder, 2022). On the other hand, in many studies, the positive value of negative affect has recently been more thoroughly emphasized in both academic and clinical psychology (Wong, 2019), including, as pointed out, feelings of regret (Saffrey et al., 2008). In McAdams and Bowman’s (2001) studies of the development of narrative identity in adulthood, “redemption” stories, where relating to painful life events play a central role, offer the richest possibilities for personal growth. In line with this, a study of emerging adults found that telling life stories focusing on mortality events was related to meaning and generativity (McLean and Pratt, 2006). Mortality events can be about the person’s first time facing personal or a close other’s vulnerability, often leading to an exploration of life and death, reflecting one’s place in the world, or reevaluating one’s values. Also, in studies of posttraumatic growth, incidents that are in themselves overwhelmingly painful are shown to give rise to an increased existential awareness that makes a richer and more developed identity possible (Pals and McAdams, 2004). From a Heideggerian perspective, Stolorow (2003) discusses the possibility that the experience of trauma can open an existential awareness that makes one recognize existential guilt, and where one takes one step back from das Man. Existential anxiety and existential guilt may have central roles in identity formation.

## The unlived life might be an ordinary one – avoiding the perfectionist trap

The psychology of personal freedom and responsibility is strongly connected to cultural norms, values, and ideologies that have found a new shape in recent decades. How can existential guilt be related to current conceptions of self-improvement and perfectionism?

In neoliberal constructions of self and its strong emphasis on individual self-reliance, the idea of finding one’s unique individual path may also, paradoxically, be felt like a demand from the outside (Foster, 2016). Impulses from humanistic psychology that can be found in the current self-help culture can also take the form of pressure toward actualizing a socially decontextualized self, where potentials are mainly seen as possible “improvements” of self-esteem, habits, or strivings for success (Madsen, 2015).

Perfectionistic tendencies have been increasing in the population in recent decades, and ideas of constant self-improvement are also a part of the perfectionistic trend, affecting identity construction (Curran and Hill, 2017). In a culture where competitive individualism plays a huge role in social networks and career and economic merits, the process of self-exploration and establishing true commitments will also be affected.

Socially prescribed perfectionism, with experience of high and often unrealistic expectations from others, is associated with increased levels of anxiety (Curran and Hill, 2017). The pop psychology idea of actualizing personal potentials, self-realization, and constant personal growth might feed this anxiety when it becomes a social norm and a criterium for success. The idea of “being the best version of yourself” can also result in identity processes where young people overanalyze their path for the future through rumination and worry and integrate perfectionism into their identity formation (Negru-Subtirica et al., 2021). Having “perfect” personal growth as a goal puts one in danger of objectivization and closure of possible choices – some lines of actions become “the right ones” independent of the person who chooses them. The idea of becoming the best version of oneself might have become a new conformist dialect of das Man speaking.

In some sense, the perfectionist version of life can be seen as a cultural norm where time needs to be used and filled as much as possible with “productive” activity as possible (Eriksen, 2001). As Taylor (2014) points out, acceleration creates a pervasive sense of anxiety rather than improving life; the future ends up seeming threatening rather than promising. In the idea of “time management,” time is handled as an object that can be manipulated (Davies and Bansel, 2005). The Heideggerian understanding of time, choices, and action marks the contrast to this way of relating to time (Heidegger, 1957). When increasing the quantitative number of activities and engagement in our lives becomes a goal in itself, finitude is also easily objectivized; death becomes more to be understood as the mere quantitative limit of years, not as the boundary of our personal life spans. Acting in ways that are true to oneself depends upon the realization that one possibility chosen means letting go of other options. In this perspective, a lifetime is not something we simply “use”; it is far more personal: Our lifetime, with our choices and the contextual chain of actions they are woven into, is part of what we are and become.

## Do our culture need a deeper insight into existential guilt?

As I have pointed out, in German and Danish, the languages of Heidegger (1957) and Kierkegaard (2017), the concept of guilt, “Schuld” and “skyld,” is not only related to transgressions but also to owing something to someone. In these conceptualizations, existential guilt is described as existential responsibility more than merely being “guilty” in the Anglo-Saxon meaning of the concept. We owe accountability to ourselves, our fellow human beings, and nature. Existential guilt, in this sense, is a call of duty as living persons and an analysis of the consequences of not doing so.

The cultural context, and the conceptualizations of selfhood, have shifted since the days of May and Yalom’s writings on existential guilt. As I already have briefly outlined, one critique of developments in current western culture highlights how neoliberal ideals of identity overemphasize individual self-reliance, and the idea of finding one’s unique individual path may also, paradoxically, be felt as a demand from the outside (Bauman, 2013; Foster, 2016). In such a condition, people will tend to blame themselves for suffering in life rather than addressing social conditions in need of change and collective actions that need to be taken.

Another critique of developments in current western culture highlight aspects that, at first glance, seem opposite to the ideals of being overly self-reliant. In what is described as “victimhood culture,” the idea is to appeal to legal authority for even minor slights or insults and dramatize a personal narrative of suffering (Campbell and Manning, 2018). In this version of selfhood, responsibility and the source of self-worth is to a more substantial degree placed outside the self, in the collective, and the society’s recognition of injustices and suffering. A contrast to victimhood culture is traditional cultures of honor. In these cultures, self-worth is attached to physical bravery and the unwillingness to be dominated by anyone. Victimhood culture is also different from the ideals of a “dignity culture,” where people are said to have dignity as a kind of inherent worth that others cannot alienate. In a dignity culture, the ideal is to be thick-skinned and less touchy than in both victimhood culture and honor culture. As a consequence of this, socialization tends to emphasize self-restraint and tolerance.

At first glance, the description of an isolated and self-contained liberal self seems incompatible with the definition of a victimhood culture where the individual most and foremost places responsibility for their life on their surroundings. As they, respectively, point to individualism and collectivism at the root of the problem, they also seem to have different political implications. However, an individual lacking binding relationships with specific others and lacking participation in an organic and developing community of equals appears to be a shared concern in both formulations of current cultural problems. Suppose we assume that both critiques of developments of selfhood in contemporary western culture have a kernel of truth. In that case, the conceptualizations of existential guilt, especially in May’s (1983) more broadly contextualized analysis, may have relevance.

The critique of the neoliberal self is primarily ambivalent and seldom antithetic toward individualism as such. However, the critics of neoliberal constructions of identity can often be vaguely nostalgic when discussing what optimal conditions of individual freedom might be. In contrast to the neoliberal ideals of atomized self-reliant individuality, existential guilt also highlights potentialities that are inherently bound to the “Midtwelt” and our life as part of relationships. A conceptualization of personal responsibility, anchored in dialog and relational being, as May (1983) outlines, and existential awareness of how finitude individualizes us, as Heidegger (1957) outlines, can illuminate some of these dilemmas.

Suppose we are to use the typologies of cultures described by Campbell and Manning (2018). In that case, the idea of existential guilt, with its emphasis on personal responsibility, can in itself be seen as rooted in dignity culture. What they describe as a victimhood culture has one crucial aspect in common with neo-liberal ideals of selfhood – a self decontextualized from personal relationships. In a victimhood culture, self-worth is achieved through appeal to legal authority or the impersonal masses on social media. The dimension of personal bonds and commitment to communities built on freedom and equality with others is lacking. As Campbell and Manning point out, legal overdependency and strong social control through the masses are also traits associated with totalitarian societies. During the second world war and the post-war era, Fromm (1941) pointed out both the dangers of pure market orientation, with its empty individualism, shallow relationships, and alienation from the community on the one hand and the “escape from freedom” into collectivistic totalitarianism on the other. The contextualization of personal responsibility offered through the concept of existential guilt might address both dangers. It provides a perspective on how personal responsibility is embedded in contexts of human relationships, nature, and the finitude, freedom, uncertainties, and suffering that is given through human existence.

## Conclusion

At a psychological level, existential guilt can wake sorrow and regret over opportunities overlooked and lost. However, most of all, it can be seen as a drive toward repair in the relationship to both oneself and the other. It takes the form of receptivity, an openness to a life not yet lived, and creative use of imagination. Existential guilt is mostly about what the next chapter in the life narrative might be and whatever time is left before death. Furthermore, this might be a chapter about an unremarkable protagonist living an ordinary life with presence and integrity in relation to self, others, and the natural world.

At an ontological level, the concept of existential guilt describes preconditions for responsibility and accountability in life choices. At a psychological level, it might also be used as a point of departure to examine an individual’s relationship to the potential given in their life and life context and, in this way, the hitherto unlived life of an individual. Heidegger highlights how recognition of finitude and individualized self-awareness is mutually dependent upon each other, and how choosing also implies letting go of possibilities. Within the framework of humanistic-existential psychology, Rollo May also highlights the dimension of human potential and its proper place concerning both self, other, and nature. As a psychological phenomenon, existential guilt can be seen as an implicit driving force in what is described as “identity achievement” within the Eriksonan approaches to identity and what Berszonsky describes as an “informational” identity style. Existential guilt can also be regarded as an antidote to perfectionist ideals for life and a call for the imperfect but meaning-oriented life. In contemporary theories of constructions of selfhood, the dangers of alienation from the community on the one hand and escape into what might become totalitarian collectivism on the other is pointed out. The concept of existential guilt offers a balanced perspective on individual responsibility and the necessity of relationships and community in personal growth across these trends.

## Author contributions

The author confirms being the sole contributor of this work and has approved it for publication.

## Conflict of interest

The author declares that the research was conducted in the absence of any commercial or financial relationships that could be construed as a potential conflict of interest.

## Publisher’s note

All claims expressed in this article are solely those of the authors and do not necessarily represent those of their affiliated organizations, or those of the publisher, the editors and the reviewers. Any product that may be evaluated in this article, or claim that may be made by its manufacturer, is not guaranteed or endorsed by the publisher.
